# Incremental Effectiveness of Emergency Vaccination Against a Varicella Outbreak at an Elementary School in Beijing, China, 2019: An Observational Cohort Study

**DOI:** 10.3390/vaccines12101184

**Published:** 2024-10-17

**Authors:** Zhiqiang Cao, Dan Zhao, Rujing Shi, Yanhong Zhao, Xiaojing Wen, Ying Ma, Xiaomei Li, Luodan Suo

**Affiliations:** 1 Department of Eexpanded Programme on Immunization, Beijing Center for Disease Prevention and Control, Beijing 100013, China; caozhiqianger@163.com (Z.C.); janedano@163.com (D.Z.);; 2 Department of Eexpanded Programme on Immunization, Haidian Center for Disease Prevention and Control, Beijing 100080, China

**Keywords:** varicella outbreak, emergency vaccination, post-exposure prophylaxis, vaccine effectiveness

## Abstract

(1) Background: The effect of varicella emergency vaccination (EV) has not been fully evaluated. (2) Methods: This was a cohort study. Participants were categorized into five groups based on their immune status: unvaccinated group, first dose as EV group, one dose no EV group, second dose as EV group, and two doses no EV group. A Cox proportional hazards model was employed to examine the association between the EV measures and the varicella incidence rate in this outbreak. (3) Results: Demographic characteristics, vaccination details, and disease onset information were 100% (918/918) collected. The crude attack rate was 44% (11/25), 8% (3/36), 11% (24/215), 3% (6/176), and 2% (8/466) among the unvaccinated group, first dose as EV group, one dose no EV group, second dose as EV group and two doses no EV group, respectively. Compared to the unvaccinated group and the one dose no EV group, the first dose varicella vaccine as EV and the second dose as EV demonstrated an incremental effectiveness of 90% (95% CI 65–97%) and 79% (95% CI 47–92%), respectively. (4) Conclusions: Both the first dose and the second dose as EV contributed to reducing the incidence rates of varicella and offered incremental vaccine effectiveness in an outbreak setting. Our study underscores the importance and benefits of initiating emergency varicella vaccination early to reduce the disease incidence rate in an elementary school setting where there was no complete coverage of the two doses of varicella vaccine and an outbreak occurred.

## 1. Introduction

Varicella, known as chickenpox, is a highly contagious viral disease that primarily affects children, causing an itchy rash with blisters and flu-like symptoms. While typically considered a mild childhood illness, varicella can lead to severe complications, especially in vulnerable populations [1]. The two-dose varicella vaccine has been reported to play an effective role in preventing the onset of varicella and reducing the severity of the disease [2,3].

Due to non-national immunization program vaccine policies, varicella vaccine coverage rates vary widely across countries and regions worldwide, ranging from below 10% to above 80% [4,5,6]. Moreover, breakthroughs of varicella could also happen in those who have received only one dose of the vaccine [4,5,7]. To better control disease transmission among institution-based populations without two-dose varicella vaccine coverage, in 2005, the Advisory Committee on Immunization Practices recommended that persons who do not have adequate evidence of immunity should receive their first or second dose of the vaccine as soon as an outbreak is identified [8].

Currently, there is limited research evaluating the effectiveness of emergency varicella vaccination following disease outbreaks, thus making it challenging to establish a reliable causal relationship between the EV measure and its impact on varicella outbreak control. Furthermore, a meta-analysis of the vaccine effectiveness of varicella EV in controlling outbreaks shows significant heterogeneity [9,10,11]. This poses a challenge for the use and evaluation of EV in the event of a varicella outbreak.

Since June 2006, Beijing has implemented a policy of providing EV for children in kindergartens and students under 15 years old in schools where cases of varicella have occurred [12]. Beijing also initiated regional varicella surveillance in 2007 to report cases in accordance with China’s class C notifiable infectious diseases requirements [13]. We conducted an observational cohort study on the impact of EV on the varicella incidence rate in a primary school in Beijing where a varicella outbreak took place.

## 2. Methods

### 2.1. Outbreak Setting

On 25 October 2019, a varicella outbreak occurred at a primary school in Haidian District, Beijing. The school had a total of 967 students in grades 1 to 6. The school consisted of a U-shaped three-story classroom building, with 15 classrooms on each floor, each classroom approximately 30 square meters in size. Each grade had 4–5 classes, with 27–33 students per class. Additionally, there were extracurricular classes for students in grades 2–6 every week, where students attended classes across different grades and classes. The last reported case occurred on 22 November, and within the subsequent maximum incubation period of 21 days, no further cases were reported.

### 2.2. Outbreak Control Measures

Students with varicella need to stay home from school until all the vesicles have crusted over. After school each day, classrooms are disinfected and surfaces in the restrooms, corridors, desks and chairs, doorknobs, and other shared items are wiped and disinfected by designated personnel. Each classroom ensures ventilation by keeping the windows open. All extracurricular classes were suspended on 7 November.

From 30 October to 10 December, children without an immunization history were given one dose of varicella vaccine for free, and children with one dose of immunization history receive one dose of varicella vaccine at their own expense, as recommended. All vaccinations were administered on a voluntary basis. The vaccine used in this study was the live-attenuated varicella vaccine by Changchun Keygen Biological Products Co., Ltd., Changchun, China.

### 2.3. Study Design and Data Collection

This was an observational cohort study. According to the requirements of the related regulations of government, Beijing has provided emergency vaccination for children in kindergartens and students under 15 years old in primary and secondary schools where cases of varicella have occurred since 2006 [12]. Beijing also initiated regional notifiable varicella surveillance in 2007 by mandating medical institutions to report cases in accordance with China’s class C notifiable infectious diseases requirements [13]. A local varicella surveillance system was also established the same year.

The data collection process was a requirement of government regulations. The surveillance system in Beijing mandates that all clinical practitioners report varicella cases electronically through the National Notifiable Disease Reporting System, a web-based computerized reporting system, to the local health department within 24 h. The analysis was limited to students who were present at school during the outbreak, had no previous history of varicella, and whose parents consented to a phone interview. Local medical staff collected demographic characteristics and follow-up data on rash illnesses through questionnaire surveys. Information on children’s past history of varicella was collected through class WeChat groups by gathering information from the parents. Children’s immunization history and EV information were obtained from the Beijing’s Immunization Information System and school vaccination records. The onset time and clinical manifestations of children with varicella incidents were collected from the parents of the children through phone interviews, including the fever severity, number of rashes, duration of rashes, and isolation period. All these procedures listed above were completed within one week after the end of the outbreak.

The current research analysis is an observational study utilizing routinely collected surveillance data. Whether to receive emergency vaccination is not an intervention actively provided by researchers but rather a post-analysis of the spontaneous behavior of the research subjects. All data collection was performed with parental consent, and data were anonymized for processing and analysis. This observational research ensures public benefits and does not violate the rights of the participants.

### 2.4. Study Definitions

The definition of cases included fever and rash occurrences at the school during the varicella outbreak, ruling out other causes. A laboratory diagnosis using the Polymerase Chain Reaction method was conducted on the vesicular fluid of early cases. The immunization history referred to the doses of varicella vaccine administered to students prior to the outbreak.

According to previous researches, individuals developed an immune response 4–7 days after varicella vaccination [14,15,16,17]. It was assumed that it takes at least 4 days after vaccination to induce sufficient antibodies against varicella-zoster virus infection, and students transitioned to the corresponding immune status 4 days after EV. Based on the immunization history and whether EV was given, students were categorized into 5 immunization statuses: no immunization history and no EV (0 + 0), no immunization history but received 1 dose of EV (0 + 1), one dose immunization history and no EV (1 + 0), one dose immunization history and received 1 dose of EV (1 + 1), and two doses immunization history with no EV (2 + 0).

### 2.5. Statistical Analysis

An epidemiological chart was used to describe the number of varicella cases and the cumulative rate of EV during the outbreak. A descriptive analysis was employed to show the baseline characteristics of study population and disease spectrum under different vaccine immunization statuses. Chi-square tests and Fisher’s exact tests were utilized to compare proportions of categorical variables based on the vaccination status, while Wilcoxon rank-sum tests were used to compare medians of continuous variables, assuming non-normal distributions.

The incidence rate of varicella was calculated by person–time according to different immunization statuses. Multivariate Cox proportional hazards models were applied to assess the effectiveness of the varicella vaccination on the disease incidence rate among those students. The EV status was treated as time-dependent covariate. For students who received a first dose as EV or second dose as EV, disease exposure was quantitatively assessed separately for the time period before and after their transition to the vaccinated status. To account for confounding factors, baseline characteristics such as sex, grade, and the interval between the immunization history and risk exposure were included as control variables in the multivariate analysis. The hazard ratio (HR) was estimated for the relative risks of the incidence rate of breakthrough varicella among EV recipients compared with those who were unvaccinated or received only one dose prior to EV. The incremental effectiveness of EV was calculated using Yule and Greenwood’s formula: 1 minus the relative risk [18].

There were two sensitivity analyses conducted to further examine the incremental effectiveness of EV: (1) limited to students with classroom exposure and (2) limited to students with less than a 2-dose immunization history.

Statistical significance was defined as a two-sided *p* value less than or equal to 0.05. All statistical analyses were conducted using R software (version 4.3.0).

## 3. Results

### 3.1. Baseline Characteristics of the Study Population

**Figure 1:**
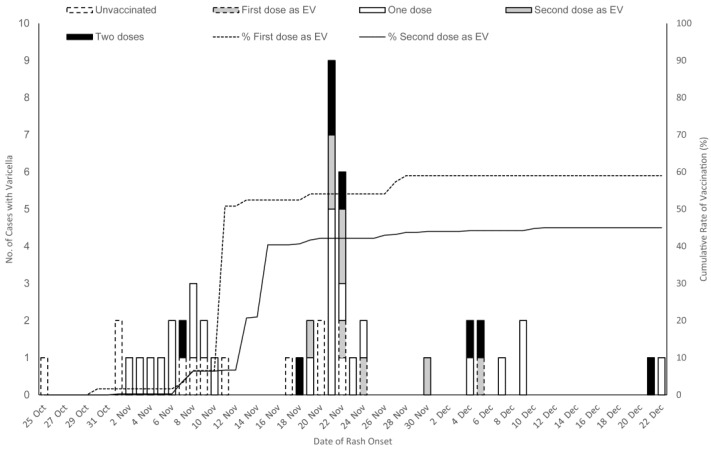
Number of cases by rash onset date and the cumulative rate of students receiving a first or second dose of the vaccine as an emergency vaccination (EV). Each peak on the epidemic curve is approximately 2 weeks apart, aligning with the recognized incubation period for varicella.

Among all 968 students, 46 students with a history of varicella before the outbreak, and 3 students who were on leave or had transferred out were excluded from the analysis, resulting in a total of 918 students included in the analysis. Personal information, vaccination details, and disease onset information were 100% collected. Table 1 presented the baseline characteristics of these 918 students. Of the unvaccinated pre-EV students, 71% (36/61) received the first dose as EV. Of the one dose pre-EV students, 45% (176/391) received the second dose as EV. Students were divided into four groups based on varying varicella immune conditions: 25 in the unvaccinated group, 36 in the first dose as EV group, 215 in the one dose no EV group, 176 in the second dose as EV group, and 466 in the two dose group. Their median age was 9.0 years, 53.4% (490/918) were male, 18.6% (171/918) were in grade 2, and the interval between the immunization history and risk exposure was 5.3 (2.4–7.9) years. The PCR method detected a 100% positivity rate from the initial 13 cases by 8 November (Figure 1). The total attack rate was 5.7% (52/918).

### 3.2. EV Measures

All vaccinations were administered on a voluntary basis. For those children who had a history of receiving no dose of the varicella immunization, the earliest EV began on 30 October. As of 11 November, 42.2% (27/64) of the unvaccinated target population had completed the first dose of EV. For those children who had a history of receiving one dose of the varicella immunization, the earliest EV began on 1 November. As of 15 November, 40.4% (158/391) of the one-dose target population had completed the second dose of EV.

By the end of the epidemic, a total of 212 individuals in the entire school received EV. The EV rate for individuals without immunity and those with one dose of immunization was 59.0% (36/61) and 45.0% (176/391), respectively, as shown in Table 1.

### 3.3. Disease Spectrum

All 52 cases presented with rash symptoms. Among them, 40.4% (21/51) experienced fever symptoms, with the highest temperature reaching 40 °C, 11.5% (6/51) had more than 50 lesions, the median time of rash duration was 11 days, and the median time of isolation duration was 13 days. No complications were reported in any of the cases. There were no statistically significant differences in the degree of fever, number of lesions, duration of rash, and isolation time among children with different immune statuses, as detailed in Table 2.

### 3.4. Incremental Effectiveness of EV for the Varicella Incidence Rate among All the Students

Table 3 presents both the unadjusted and adjusted incremental effectiveness of the first dose as EV and the second dose as EV on the varicella incidence rate among all the participants. A total of 918 students and 53,360 person-days were recorded during the varicella outbreak period. The overall incidence rate was 9.7 per 10,000 person-days among this study. The varicella incidence rates were 105.8, 14.4, 20.0, and 5.8 per 10,000 person-days for unvaccinated students, those who received the first dose as EV, students who had only one dose without EV, and those who received the second dose as EV, respectively.

In both univariate and multivariate models, the incremental effectiveness of the EV on the varicella incidence rate remained consistent. Multivariate analyses indicated that students in the first dose as EV group had lower rates of varicella incidence (aHR 0.10, 95% CI 0.03–0.35) when compared with the unvaccinated group. The incremental effectiveness of the first dose as EV was 90% (95% CI 65–97%). Similarly, students in the second dose as EV group had lower rates of varicella incidence (aHR 0.21, 95% CI 0.08–0.53) when compared with the one dose no EV group. The incremental effectiveness of the second dose as EV was 79% (95% CI 47–92%).

### 3.5. Incremental Effectiveness of EV for the Varicella Incidence Rate among Students with Classroom Exposure

Table 4 presents both the unadjusted and adjusted incremental effectiveness of the first dose as EV and the second dose as EV on the varicella incidence rate among students with classroom exposure. Multivariate analyses indicated that students in the first dose as EV group had lower rates of varicella incidence (aHR 0.12, 95% CI 0.03–0.45) when compared with the unvaccinated group. The incremental effectiveness of the first dose as EV was 88% (95% CI 55–97%). Similarly, students in the second dose as EV group had lower rates of varicella incidence (aHR 0.18, 95% CI 0.07–0.45) when compared with the one dose no EV group. The incremental effectiveness of the second dose as EV was 82% (95% CI 55–93%).

### 3.6. Incremental Effectiveness of EV for the Varicella Incidence Rate among Students with Less than a Two-Dose Immunization History

Supplemental Table S1 presents both the unadjusted and adjusted incremental effectiveness of the first dose as EV and the second dose as EV on the varicella incidence rate among students with less than a two-dose immunization history. Similar to the analysis results for the entire population, the incremental effectiveness of the first and second doses as EV was 91% (95% CI 66–97%) and 80% (95% CI 49–92%), respectively, among students with less than a two-dose immunization history.

### 3.7. Vaccine Effectiveness without EV for the Varicella Incidence Rate

Table 3 showed that students in the one dose no EV group and two doses no EV group had lower rates of varicella incidence (aHR 0.22, 95% CI 0.11–0.46; aHR 0.03, 95% CI 0.01–0.08) compared to the unvaccinated group. The effectiveness of the one dose no EV and two doses no EV was 78% (95% CI 45–91%) and 97% (95% CI 92–99%), respectively.

### 3.8. Characteristics of the Participants in Two Doses No EV Group Categorized by the Onset Status

Supplemental Table S2 presents the baseline characteristics of the 466 students in the two dose no EV group categorized by the onset status. Among the eight breakthrough cases, the median age was 9.6 years, 75.0% (6/8) were male, and the interval between the immunization history and risk exposure was 4.4 years. Among the 458 non-cases, the median age was 8.0 years, 52.4% (240/458) were male, and the interval between immunization history and risk exposure was 2.9 years.

## 4. Discussion

This study demonstrates the benefits of the incremental effectiveness of the first and second doses of the varicella vaccine in reducing the disease incidence rate in a school where there was no complete coverage of the two doses of varicella vaccine and an outbreak occurred. It was revealed that both the first dose of varicella vaccine as EV and the second dose as EV contributed to reducing the incidence rates of varicella and offered incremental vaccine effectiveness of 90% and 79%, respectively, in an outbreak setting. Our data first show that emergency varicella vaccination measures are effective in reducing the disease incidence rate both within the whole student population and the classroom-exposed students in schools.

All our sensitivity analysis results indicated that the causal relationship between emergency vaccination and protective effects was stable. It was demonstrated that the incremental effectiveness of EV was significant, but limited among students who had classroom exposure. It should be noted that grade 1 students mostly belonged to the population without classroom exposure in this study. The results of the incremental effectiveness of EV were robust, whether excluding the grade 1 students or the two doses no EV group of students.

Our findings were similar to those of previous studies that demonstrated a significant incremental effectiveness of the first and second doses of the varicella vaccine for outbreak control at schools in Philadelphia and Shanghai [17,19,20]. Meanwhile, some studies suggested that the vaccine effectiveness of varicella EV in schools was influenced by various factors. For instance, the vaccine effectiveness of the second dose of the varicella vaccine as post-exposure prophylaxis in affected classrooms with high vaccine uptake was higher compared to classrooms with lower vaccine uptake [9,19,21]. The adjusted vaccine effectiveness was also higher in students who received the second dose of the varicella vaccine within 3 days of exposure, as opposed to those who received it more than 3 days after exposure [9,19,21]. This suggests that the stability of vaccine effectiveness in schools is considered uncertain, making it difficult to measure the benefits of emergency vaccination and guide practical actions. However, we hold the view that the heterogeneity of vaccine effectiveness in different studies may be more fundamentally related to the differences in exposure risk between the EV group and the non-EV group. In other words, the imbalance of the varicella infection exposure risk between the two groups could lead to confounding bias when assessing the protection effectiveness. We used the incidence density to adjust for the varying exposure risks caused by differences in observation times. Furthermore, the exposure risks before vaccination were included in the non-EV group, akin to the principle of self-matching, which enhanced the balance of exposure risks between this two groups.

The study demonstrates that varicella EV offers practical public health benefits. Varicella outbreaks usually account for a high proportion of school outbreaks, involving a large number of cases, but many regions lack unified immunization and emergency vaccination strategies [4,5,6,22]. For individual post-exposure prophylaxis, the WHO recommends vaccination as soon as possible, within 3–5 days post-exposure, which can be effective in preventing disease [23]. However, it is difficult to determine whether individual students have been exposed in classrooms due to chickenpox having a latent period, especially when cases have just emerged in the school. Our study showed that administering varicella emergency vaccination to school-exposed students during an outbreak can also provide public health benefits. Additionally, there is a scarcity of data to evaluate the effectiveness of emergency vaccination implemented more than five days after an outbreak occurs in controlling outbreaks for public population health. This study provides direct evidence that both the first dose and the second dose as EV implemented more than five days after an outbreak occurs contributed to lower rates of varicella incidence and offered incremental vaccine effectiveness in an outbreak setting.

Additionally, routine varicella vaccination demonstrates significant protective effectiveness. We have assessed the effectiveness of varying varicella vaccination statuses in reducing the incidence of this disease to showcase a more comprehensive vaccine group efficacy. Compared with the unvaccinated group, students with a history of either one dose or two doses of varicella vaccination displayed a significantly decreased disease incidence by 78% and 97%. Additionally, an intriguing finding in our study suggests that the first dose of EV may not achieve the same effectiveness as the two doses no EV, and the second dose as EV might achieve an effectiveness that is slightly lower than the two doses no EV. All of the above information indicates that administering two doses of varicella vaccine to eligible children is a measure that effectively prevents the onset of chickenpox at school.

However, the protective effectiveness of the routine varicella vaccination recommended in two doses may slightly decline over time. We have found that a few students who were fully vaccinated developed mild varicella. Most cases occurred more than 4 years after the second dose of vaccination, which may be longer than non-cases considering the impact of the small sample size. This varicella breakthrough may be partially explained by the normal decay of the vaccine as a preliminary analysis. The vaccine failure or the declining immune effectiveness of the varicella vaccine requires further research to better mitigate the burden of varicella disease. Given the relatively high rates of a protective effect with two doses and unlimited public resources, it is still preferable to recommend the two-dose routine vaccination for the child population.

Several limitations of this study warrant attention. First, this observational cohort study may have omitted variables related to varicella incidence, such as genetic predisposition and individual immune status. These variables could lead to confounding bias when assessing the vaccine effectiveness, particularly under conditions of an imbalanced distribution. Efforts were made to measure and include in the model any covariates that could influence the incremental effectiveness of EV, and we did not observe whether the inclusion of any known covariates had an impact on our results. Second, the assumption that it takes 4 days to transition to the corresponding immune status underestimates the effectiveness of varicella vaccination. Third, the vaccine used in this study was the live-attenuated varicella vaccine from Changchun Keygen Biological Products Co., Ltd. Different types of vaccines may exhibit variations in vaccine effectiveness.

## 5. Conclusions

Our study demonstrated that both the first dose and the second dose as EV contributed to lower rates of varicella incidence and offered incremental vaccine effectiveness after exposure to varicella in an outbreak setting. This study establishes a reliable causal relationship between the EV measure and its impact on varicella prophylaxis using individual-level information. Our study underscores the importance and benefits of initiating emergency varicella vaccination early to reduce the disease incidence rate in a school where there was no complete coverage of the two doses of varicella vaccine and an outbreak occurred.

## Figures and Tables

**Table 1:** Baseline characteristics of participants categorized by immune status.

Variable	Total	Unvaccinated	First Dose as EV	One Dose No EV	Second Dose as EV	Two Doses No EV
Total	918 (100.0)	25 (100.0)	36 (100.0)	215 (100.0)	176 (100.0)	466 (100.0)
Age	9.0 (7.6–10.6)	9.5 (8.5–10.1)	9.1 (7.4–9.9)	10.2 (9.2–11.1)	9.3 (7.9–10.7)	8.0 (7.0–10.2)
Sex						
Female	428 (46.6)	10 (40.0)	16 (44.4)	91 (42.3)	91 (51.7)	220 (47.2)
Male	490 (53.4)	15 (60.0)	20 (55.6)	124 (57.7)	85 (48.3)	246 (52.8)
Grade						
1	160 (17.4)	0 (0.0)	5 (13.9)	5 (2.3)	18 (10.2)	132 (28.3)
2	171 (18.6)	5 (20.0)	7 (19.4)	8 (3.7)	35 (19.9)	116 (24.9)
3	138 (15.0)	4 (16.0)	6 (16.7)	36 (16.7)	33 (18.8)	59 (12.7)
4	152 (16.6)	9 (36.0)	14 (38.9)	53 (24.7)	35 (19.9)	41 (8.8)
5	143 (15.6)	3 (12.0)	1 (2.8)	61 (28.4)	19 (10.8)	59 (12.7)
6	154 (16.8)	4 (16.0)	3 (8.3)	52 (24.2)	36 (20.5)	59 (12.7)
Classroom Exposure						
Yes	558	20(80.0)	27 (75.0)	140 (65.1)	124 (70.5)	247 (53.0)
No	360	5(20.0)	9 (25.0)	75 (34.9)	52 (29.5)	219 (47.0)
Varicella Incidence						
Yes	52 (5.7)	11 (44.0)	3 (8.3)	24 (11.2)	6 (3.4)	8 (1.7)
No	866 (94.3)	14 (56.0)	33 (91.7)	191 (88.8)	170 (96.6)	458 (98.3)
Interval between the Immunization History and Risk Exposure, years	5.3 (2.4–7.9)	/	/	8.6 (7.3–9.6)	7.6 (5.5–8.9)	3.0 (1.3–5.0)

Data are presented as Nos. (%) or medians (interquartile ranges).

**Table 2:** Spectrum of illness for varicella cases associated with outbreaks based on the vaccination status.

Clinical Characteristic	Total *n* = 52	Unvaccinated *n* = 11	First Dose as EV *n* = 3	One Dose No EV *n* = 24	Second Dose as EV *n* = 6	Two Doses No EV *n* = 8	*p*
Fever	21 (40.4)	6 (54.6)	0 (0.0)	8 (33.3)	2 (33.3)	5 (62.5)	0.306
>50 lesions	6 (11.5)	3 (27.3)	0 (0.0)	1 (4.52)	2 (33.3)	0 (0.0)	0.073
Rash duration	11 (9–14)	14 (10–17)	11 (5–11)	12 (8–13)	9 (7–14)	11 (5–14)	0.281
Isolation duration	13 (10–17)	15 (13–18)	11 (7–17)	14 (10–16)	12 (7–15)	13 (13–15)	0.387

Data are presented as Nos. (%) or medians (interquartile ranges).

**Table 3:** Incremental effectiveness of EV for the varicella incidence rate among all the students.

Varicella Vaccination Status	Number	Cases	Follow-Up Time (Person-Days)	Incidence Rate (/10,000 Person-Days)	HR (95% CI)	*p*	aHR * (95% CI)	*p*
Total	918	52	53,360	9.7				
Unvaccinated	25	11	1040	105.8	ref		ref	
First Dose as EV ^#^	36	3	2085	14.4	0.13 (0.04–0.47)	0.002	0.10 (0.03–0.35)	<0.001
One Dose No EV	215	24	12,109	20.0	0.18 (0.09–0.37)	<0.001	0.22 (0.11–0.46)	<0.001
Second Dose as EV	176	6	10,376	5.8	0.05 (0.02–0.14)	<0.001	0.05 (0.02–0.13)	<0.001
Two Doses No EV	466	8	27,750	2.9	0.03 (0.01–0.07)	<0.001	0.03 (0.01–0.08)	<0.001

^#^ EV, emergency vaccination. * aHR, adjusted hazard ratio. HRs were calculated using both univariate and multivariate Cox regression analyses, and adjusted for sex, grade, classroom exposure, and the interval between the immunization history and risk exposure. Compared with the one dose no EV group, students in the second dose as EV group had lower rates of varicella incidence (aHR 0.21, 95% CI 0.08–0.53). Vaccine effectiveness equals 1 minus HR. Compared to the unvaccinated group and one dose no EV group, the first dose varicella vaccine as EV and the second dose as EV offered an incremental effectiveness of 90% (95% CI 65–97%) and 79% (95% CI 47–92%), respectively.

**Table 4:** Incremental effectiveness of EV for the varicella incidence rate among students with classroom exposure.

Varicella Vaccination Status	Number	Cases	Follow-Up Time (Person-Days)	Incidence Rate (/10,000 Person-Days)	HR (95% CI)	*p*	AHR * (95% CI)	*p*
Total	558	52	31,760	16.4				
Unvaccinated	20	11	740	148.6	ref.		ref.	
First Dose as EV ^#^	27	3	1545	19.4	0.12 (0.03–0.44)	0.001	0.12 (0.03–0.45)	0.002
One Dose No EV	140	24	7609	31.5	0.20 (0.10–0.42)	<0.001	0.25 (0.12–0.53)	<0.001
Second Dose as EV	124	6	7256	8.3	0.05 (0.02–0.15)	<0.001	0.04 (0.02–0.12)	<0.001
Two Doses No EV	247	8	14,610	5.5	0.04 (0.01–0.09)	<0.001	0.03 (0.01–0.09)	<0.001

^#^ EV, emergency vaccination. * aHR, adjusted hazard ratio. HRs were calculated using both univariate and multivariate Cox regression analyses, and were adjusted for sex, grade, class, and the interval between the immunization history and risk exposure. Compared with the one dose no EV group, students in the second dose as EV had lower rates of varicella incidence (aHR 0.18, 95% CI 0.07–0.45). Vaccine effectiveness equals 1 minus HR. Compared to the unvaccinated group and one dose no EV group, the first dose varicella vaccine as EV and the second dose as EV offered incremental effectiveness of 88% (95% CI 65–97%) and 82% (95% CI 55–93%), respectively.

## Data Availability

The data presented in this study are available upon reasonable request from the corresponding author.

## Supplementary Materials

The following supporting information can be downloaded at https://www.mdpi.com/article/10.3390/vaccines12101184/s1, Table S1: Incremental effectiveness of EV for varicella incidence rate among students with less than a 2-dose immunization history; Table S2: Characteristics of the participants in two doses no-EV group categorized by onset status.
