# Supplementary material for: Incremental Effectiveness of Emergency Vaccination Against a Varicella Outbreak at an Elementary School in Beijing, China, 2019: An Observational Cohort Study
Source: Vaccines (Basel). 2024 Oct 17;12(10):1184. doi: 10.3390/vaccines12101184 (PMC11512427; doi:10.3390/vaccines12101184)
Supplement: Supplementary file 1 [file vaccines-12-01184-s001.zip › vaccines-3246787-supplementary.pdf]

Table S1. Incremental effectiveness of EV for varicella incidence rate among students with less than a 2-dose immunization history.

Varicella Vaccination Status	Number	Cases	Follow-Up Time (Person-Days)	Incidence Rate (/10000 Person-Days)	HR (95% CI)	*p*	aHR* (95% CI)	*p*
Total	452	44	25610	17.2				
Unvaccinated	25	11	1040	105.8	ref		ref	
First Dose as EV#	36	3	2085	14.4	0.13 (0.04–0.48)	0.002	0.09 (0.03–0.34)	<0.001
One Dose No EV	215	24	12109	20.0	0.19 (0.09–0.38)	<.001	0.22 (0.11–0.47)	<0.001
Second Dose as EV	176	6	10376	5.8	0.05 (0.02–0.15)	<.001	0.05 (0.02–0.12)	<0.001

#EV, emergency vaccination. *aHR, adjusted hazard ratios. HRs were calculated using both univariate and multivariate Cox regression analyses, adjusted for sex, grade, class and interval between immunization history and risk exposure. Compared with the one dose no EV group, students of the second dose as EV had lower rates of varicella incidence (aHR 0.20, 95% CI 0.08–0.51). Vaccine effectiveness equals 1 minus HR. Compared to the unvaccinated group and one dose no EV group, the first dose varicella vaccine as EV and the second dose as EV offered incremental effectiveness of 91% (95% CI 66%–97%) and 80% (95% CI 49%–92%), respectively.

Table S2. Characteristics of the participants in two doses no-EV group categorized by onset status.

	Total *n* = 466	Cases *n* = 8	Non-Cases *n* = 458
Age	9.0 (7.6–10.6)	9.6 (7.9–10.7)	8.0 (7.0–10.2)
Sex			
Female	220 (47.2)	2 (25.0)	218 (47.6)
Male	246 (52.8)	6 (75.0)	240 (52.4)
Interval between the Immunization History and Risk Exposure, years	3.0 (1.3–5.0)	4.4 (3.7–6.3)	2.9 (1.3–5.0)
